# Intracellular virus sensor MDA5 exacerbates vitiligo by inducing the secretion of chemokines in keratinocytes under virus invasion

**DOI:** 10.1038/s41419-020-2665-z

**Published:** 2020-06-12

**Authors:** Tongtian Zhuang, Xiuli Yi, Jianru Chen, Pan Kang, Xuguang Chen, Jiaxi Chen, Tingting Cui, Yuqian Chang, Zhubiao Ye, Qingrong Ni, Yinghan Wang, Pengran Du, Baizhang Li, Ling Liu, Zhe Jian, Kai Li, Tianwen Gao, Shuli Li, Chunying Li

**Affiliations:** Department of Dermatology, Xijing Hospital, Fourth Military Medical University, No 127 of West Changle Road, Xi’an, Shaanxi 710032 China

**Keywords:** Autoimmunity, Chemokines, Infection, RIG-I-like receptors

## Abstract

Vitiligo is a disfiguring disease featuring chemokines-mediated cutaneous infiltration of autoreactive CD8^+^ T cells that kill melanocytes. Copious studies have indicated that virus invasion participates in the pathogenesis of vitiligo. *IFIH1*, encoding MDA5 which is an intracellular virus sensor, has been identified as a vitiligo susceptibility gene. However, the specific role of MDA5 in melanocyte death under virus invasion is not clear. In this study, we first showed that the expression of anti-CMV IgM and MDA5 was higher in vitiligo patients than healthy controls. Then, by using Poly(I:C) to imitate virus invasion, we clarified that virus invasion significantly activated MDA5 and further potentiated the keratinocyte-derived CXCL10 and CXCL16 which are the two vital chemokines for the cutaneous infiltration of CD8^+^ T cells in vitiligo. More importantly, IFN-β mediated by the MDA5-MAVS-NF-κB/IRF3 signaling pathway orchestrated the secretion of CXCL10 via the JAK1-STAT1 pathway and MDA5-meidiated IRF3 transcriptionally induced the production of CXCL16 in keratinocytes under virus invasion. In summary, our results demonstrate that MDA5 signaling orchestrates the aberrant skin immunity engaging in melanocyte death via mediating CXCL10 and CXCL16 secretion, which supports MDA5 as a potential therapeutic target for vitiligo under virus invasion.

## Introduction

Vitiligo is a disfiguring disease characterized by melanocyte death which is caused by autoreactive melanocyte-specific CD8^+^ T cells with the attraction of excessive chemokines^1,2^, which underlines the autoimmunity dominantly participates in the pathogenesis of vitiligo. Multiple studies showed that vitiligo is caused by specific environmental factors in the population with genetic predisposition^3^. Virus, as an important environmental factor, has been determined to play profound parts in the pathogenesis of many autoimmune diseases including type 1 diabetes^4–6^, rheumatic arthritis^7–9^, and autoimmune thyroid disease^10,11^. These autoimmune diseases all occur with increased frequency in vitiligo patients^12,13^. In vitiligo paraffin-embedding tissues, the detective rate of CMV DNA is markedly higher than that in healthy controls (38% vs 0)^14^. Besides, the case-control study based on the large sample size determines that hepatitis C virus (HCV)^15^ is associated with vitiligo. Additionally, A strong causative link between turkey herpesvirus and vitiligo has also been established in the Smyth line chicken^16^. Moreover, many cases embody the correlation between virus infection and vitiligo^17–22^, particularly the inflammatory raised borders in vitiligo with HCV infection^19^, the reactivation of herpes simplex virus in lesions of vitiligo after excimer laser treatment^20^ and vitiligo occurrence after the resolution of drug-induced hypersensitivity syndrome which is characterized by sequential reactivations of herpes viruses^21,22^. It is noted that interferon-induced helicase C domain 1 (*IFIH1*), encoding melanoma differentiation-associated 5 protein (MDA5) which could sense the invasion of virus, has been identified as a susceptible gene for vitiligo by several genome-wide association studies (GWAS)^23–25^. However, the specific role of MDA5 in the pathogenesis of vitiligo under virus invasion is not clear.

As a pathogen pattern recognition receptor (PRR), MDA5 could sense the virus replication intermediate dsRNA derived from no matter DNA or RNA virus^26,27^, and Poly(I:C) is an analog of this kind of dsRNA^28^. After sensing invasion of viruses, MDA5 triggers potent signaling cascade to eradicate virus by initiating the type I interferon (IFNs)^29^. Meanwhile, the inappropriate activation of MDA5 increases the risk of autoimmune diseases^5,30–33^. Upon activated, MDA5 could exploit the longer dsRNA (>1 kb) as a scaffold to induce the polymerization of the adaptor mitochondrial antiviral signaling protein (MAVS), a protein anchored in mitochondrial outer membrane^34–36^. Polymerization of MAVS triggers a signaling cascade culminating in the activation of the interferon-regulating factor 3 (IRF3) and the nuclear factor-kappa B (NF-κB)^37,38^. These transcription factors induce the type I and III IFNs, as well as other cytokines or chemokines^39–42^.

In vitiligo, the excessive CXCL10 (chemokine (C-X-C motif) ligand 10)^43,44^ and CXCL16^45^ in epidermis have been established to play key roles in the skin trafficking of autoreactive melanocyte-specific CD8^+^ T cells^1^, which has been recognized as the main cause of melanocyte death in vitiligo. Thus, we hypothesized that intracellular virus sensor MDA5 could exacerbate the melanocyte death in vitiligo by inducing the section of chemokines from keratinocytes under virus invasion.

In the present study, we initially manifested that the expression of MDA5 and anti-CMV IgM was upregulated in some progressive vitiligo, which was accompanied by the increment of the mRNA levels of CXCL10, CXCL16 in the epidermis and the infiltration quantity of CD8^+^ T in the skin tissues. Then, we clarified that virus invasion significantly activated MDA5 and further potentiated the secretion of CXCL10 and CXCL16 in keratinocytes. Mechanistically, we revealed that IFN-β mediated by the MDA5-MAVS-NF-κB/IRF3 signaling pathway orchestrated the secretion of CXCL10 via the JAK1-STAT1 pathway and MDA5-meidiated IRF3 transcriptionally induced the production of CXCL16 in keratinocytes under virus invasion. Therefore, we supported the MDA5 signaling pathway as a promising therapeutic target in vitiligo under virus invasion.

## Materials and methods

### Patients and samples

The peripheral blood samples and skin samples of vitiligo were collected from patients given a diagnosis of vitiligo at a progressive stage according to clinical manifestation in the Department of Dermatology, Xijing Hospital of the Fourth Military Medical University. All subjects had not received any systematic or topical therapy within 3 months before serum and skin samples collection. The control blood samples were collected from age- and sex- matched voluntary staffs working in Xijing hospital and the control skin samples collected from the age- and sex- matched subjects during plastic surgery in Xijing hospital. The skin samples of atopic dermatitis and psoriasis were collected from the in-patient given a diagnosis of atopic dermatitis and psoriasis respectively in the Department of Dermatology, Xijing Hospital of the Fourth Military Medical University. All participants gave their written informed consents according to the Declaration of Helsinki and approval from Clinical Research Ethics Committee of Xijing Hospital, Fourth Military Medical University. All serum samples were stored at -80 °C without repeated freezing and thawing. All skin samples used for immunofluorescence assay were embedded in paraffin at 4 °C. The total protein for Western-blot assay was extracted from fresh skin samples immediately and was stored at −20 °C. The total RNA for qRT-PCR assay was extracted from fresh skin samples and reverse-transcribed to cDNA and stored at −20 °C. (The numbers of subjects used for each assay were listed in Supplementary Table 1).

### Cytomegalovirus IgM detection

The cytomegalovirus IgM detection enzyme-linked immunosorbent assay (ELISA) kit (Abcam, ab108725, USA) was used to detect the anti-CMV IgM in serum according to the manufacturer’s instructions. The details are in the Supplementary methods.

### Virus invasion simulation

Poly(I:C) is a synthetic analog of double-stranded RNA (dsRNA), a molecule pattern associated with viral infection. The Poly(I:C) used in this article is Poly(I:C)/LyoVec^TM^ (Invivogen, USA) which are performed complexes between Poly(I:C) and the transfection reagent LyoVec^TM^ which can ensure that it functions in cytosol. Combined with the characteristic that they have an average size of 1.5–8 kb, these complexes could imitate the virus infection and further be sensed by MDA5.

### Cell culture

Normal Human keratinocytes (NHKs) were isolated from healthy subjects and cultured in keratinocyte-SFM (Gibco, USA). Cells were grown to 80% confluency and used in 3–5 passages. A human keratinocyte cell line was purchased from American Tissue Culture Collection (ATCC) and cultured in Modified Medium RPIM 1640 with 10% FBS and L-glutamine (Invitrogen, USA). Poly(I:C) (Invivogen, USA) was added to culture at different concentrations (0.3 μg/ml, 0.6 μg/ml) in dose-dependent assays and at 0.6 μg/ml in function and signaling pathway exploration assays. For experiments, supernatant was harvested for ELISA, and the cells were processed for quantitative real-time PCR (qRT-PCR), Western-blot and immunofluorescence assay at the indicated time points.

### Annexin V-PE/7AAD apoptosis assay

NHKs were plated into 6-well plates at the density of 3 × 10^5^ cells/well and treated with Poly(I:C) within 1.5 μg/ml for 24 h. Cell apoptosis was detected by using the Annexin V-PE/7-AAD kit (MultiSciences, China). Annexin V-PE and 7AAD fluorescence were measured using flow cytometry (Beckman Coulter, USA) and analyzed with Expo32 software (Beckman Coulter, USA).

### Immunofluorescence assay

For skin samples embedded with paraffin of progressive vitiligo patients with positive or negative anti-CMV IgM and healthy controls, the samples were deparaffinized and boiled in antigen retrieval solution (PH = 9.0). Next, the sections were incubated with the normal goat serum (BosterBio, USA) for 1 h after 3 times washing with PBS. Then, incubated with the primary antibody (rabbit monoclonal anti-MDA5, Abcam, ab126630, USA; rabbit polyclonal anti-IFN-β, Proteintech, 27506-1-AP, China; rabbit monoclonal anti-CD8α, Abcam, ab108343, USA) overnight at 4 °C. After washing, the secondary antibody (goat anti-rabbit IgG-Alexa Fluor 488 Conjugated, zhuangzhiBIO, China) was used for 1 h at room temperature in dark. The cell nuclei were stained with Hoechst 33258 (Sigma-Aldrich, German) for 10 min in dark. Immunofluorescence analysis was applied with laser scanning confocal microscopy (Olympus, Japan).

For cell immunofluorescence, the HaCaT cells or NHKs were cultured on culture plates and treated with 0.6 μg/ml Poly(I:C) for the indicated time. Then, they were fixed with 4% paraformaldehyde for 10 min. After washing, the cells were incubated with 0.1% Triton X-100 for 10 min at room temperature, and then were blocked with normal goat serum for 30 min. The following procedures were similar as the histological immunofluorescence assay with the use of primary antibodies (rabbit monoclonal anti-MDA5, Abcam, ab126630, USA; mouse monoclonal anti-MAVS, Abcam, ab220170, USA) and the secondary antibodies (goat anti-rabbit IgG-Alexa Fluor 488 Conjugated, zhuangzhiBIO, China; goat anti-mouse IgG-Cy3 Conjugated, zhuangzhiBIO, China).

### Determination of IC50 by CCK8 assay

Roughly, the 50% inhibitory concentration (IC50) of Poly(I:C) on NHKs was determined by CCK8 kit (7Sea biotech, China) according to the manufacturer’s instructions. In general, seeded in 96-well culture plates, cells were treated with 0-4 μg/ml Poly(I:C) for 24 h. Next, incubated with 100 μl fresh medium with 10 μl CCK8 solution for 2 h at 37 °C and optical density (OD) was measured at 450 nm by Model 680 Microplate Reader (Bio-Rad, USA).

### Western-blot assay

After washed and lysed, the total protein was collected and the concentration was measured with BCA Protein Assay kit (Pierce, USA). Equal amounts of protein were separated by 10% SDS-PAGE (Bio-Rad, USA) and transferred to Polyvinylidene difluoride membranes (Millipore, USA). Then the molecules were blocked with 5% non-fat dry milk for 2 h. After washed with TBST transitorily, the membranes were incubated with the corresponding primary antibodies **(**Supplementary Table 2**)** overnight at 4 °C. After washing, the membranes were incubated with secondary antibodies (horseradish peroxidase-labeled goat anti-rabbit IgG, Santa Cruz, USA; horseradish peroxidase-labeled goat anti-mouse IgG, Santa Cruz, USA) for 1 h at room temperature. The bands were detected by ChemiDocTM XRS + system (Bio-Rad, USA). As for the determination of MDA5 expression in skin tissues by Western-blot, the skin samples were soaked in Dispase at 4 °C overnight, and then we removed the dermis and subcutaneous tissues to get the epidermis tissues. The remaining procedures were similar to the above description.

### Small interfering RNA (siRNA) transfection

Cells were seeded at 3×10^5^ cells/well for 24 h before the transfection. The 6 μl corresponding siRNA (IFIH1 siRNA,5′-GCACGAGGAAUAAUCUUUATT-3′ GenePharma China; MAVS siRNA, 5′-CACAGGGUCAGUUGUAUCUTT-3′ GenePharma China; NC siRNA, 5′-UUCUCCGAACGUGUCACGUTT-3′ GenePharma China; NF-κB siRNA Santa Cruz, sc29410, USA; IRF3 siRNA Santa Cruz, sc35710, USA) and 6 μl Lipofectamine 3000 (Invitrogen, USA) were added to 250 μl 1640 medium without fetal bovine serum (FBS). After mixed evenly, it was placed at room temperature for 10 min. Then, the mixed liquid was added to each well with 2 ml FBS-containing fresh medium.

### RNA isolation and qRT-PCR

Total RNA was extracted with TRIzol (Ambion, USA) and then reverse-transcribed to cDNA using a PrimeScript RT reagent kit (Takara, Japan). qRT-PCR was performed by using SYBR Premix Ex Taq II (Takara, Japan) with the iQ5 PCR Detection System (Bio-Rad, USA). Threshold cycle (CT) values were used to calculate the fold change by the 2^-ΔΔCT^ method. The relative mRNA expression was normalized to β-actin. Primer sequences are listed in Supplementary Table 3.

### ELISA assay

Human CXCL9, CXCL12, CCL26, CCL2, IL-15, IFN-β Quantikine ELISA kits (Elabscience, China) and CXCL10, CXCL16, CCL20 DuoSet ELISA kits (R&D Systems, USA) were used to analyze cell supernatant samples, according to the corresponding manufacturer’s instructions.

### Semi-denaturing detergent agarose gel electrophoresis (SDD-AGE)

Semi-denaturing detergent agarose gel electrophoresis (SDD-AGE) was conducted according to the published protocol^36^. Crude cell lysis was resuspended in 1×sample buffer (0.5×TBE, 10% glycerol, 2% SDS, and 0.0025% bromophenol blue) and loaded onto a vertical 1.5% agarose gel (Bio-Rad, USA). After electrophoresis in the running buffer (1×TBE and 0.1% SDS) for 35 min with a constant voltage of 100 V at 4 °C, the proteins were transferred to Immobilon membrane (Millipore, USA) for immunoblotting and the following procedures are same as the Western-blot assay.

### Chromatin immunoprecipitation assay

Chromatin immunoprecipitation analysis was performed by using the ChIP (Chromatin immunoprecipitation) Assay Kit (Millipore, USA), according to the manufacturer’s instructions. Briefly, HaCaT cells were cross-linked with DNA by using 1% formaldehyde at room temperature for 10 min. After washing, cells were lysed in SDS lysis buffer. DNA was sheared to small fragments of less than 500 bp in length by means of sonication. The recovered supernatant fraction was incubated with antibodies overnight on a rotor at 48 °C. After washing, the precipitated IRF3 protein-DNA complexes were recovered by using IRF3 antibodies and protein G–Sepharose beads at 48 °C for 16 h. DNA was then purified with phenol/chloroform. A fraction was used as the PCR template (forward, 5′-CAGCCCTGGGTTCTTACCACTC-3′; reverse, 5′-AGGATGGCTGCCGAGAGGAC-3′) to detect the presence of promoter sequences between −1253 and −1338 of CXCL16. After the PCR amplification, each PCR reaction was analyzed by 2% agarose gel electrophoresis with D2000 DNA ladder (Solarbio, China).

### Statistical analysis

Each statistical analysis was performed by GraphPad Prism 7 for Windows (USA) with two-tailed Student’s unpaired t-tests or one-way analysis of variance (ANOVA). These data are conformed to the normal distribution and variance similarity between the groups that are statistically compared. These data represent as mean ± SD for at least three independent experiments. ^*^P < 0.05, ^**^P < 0.01, ^***^P < 0.001, ^#^*P* < 0.05, ^##^*P* < 0.01, ^###^*P* < 0.001, ns: not significant.

## Results

### The expression of MDA5 and anti-CMV IgM is higher in some progressive vitiligo patients

**Fig. 1:**
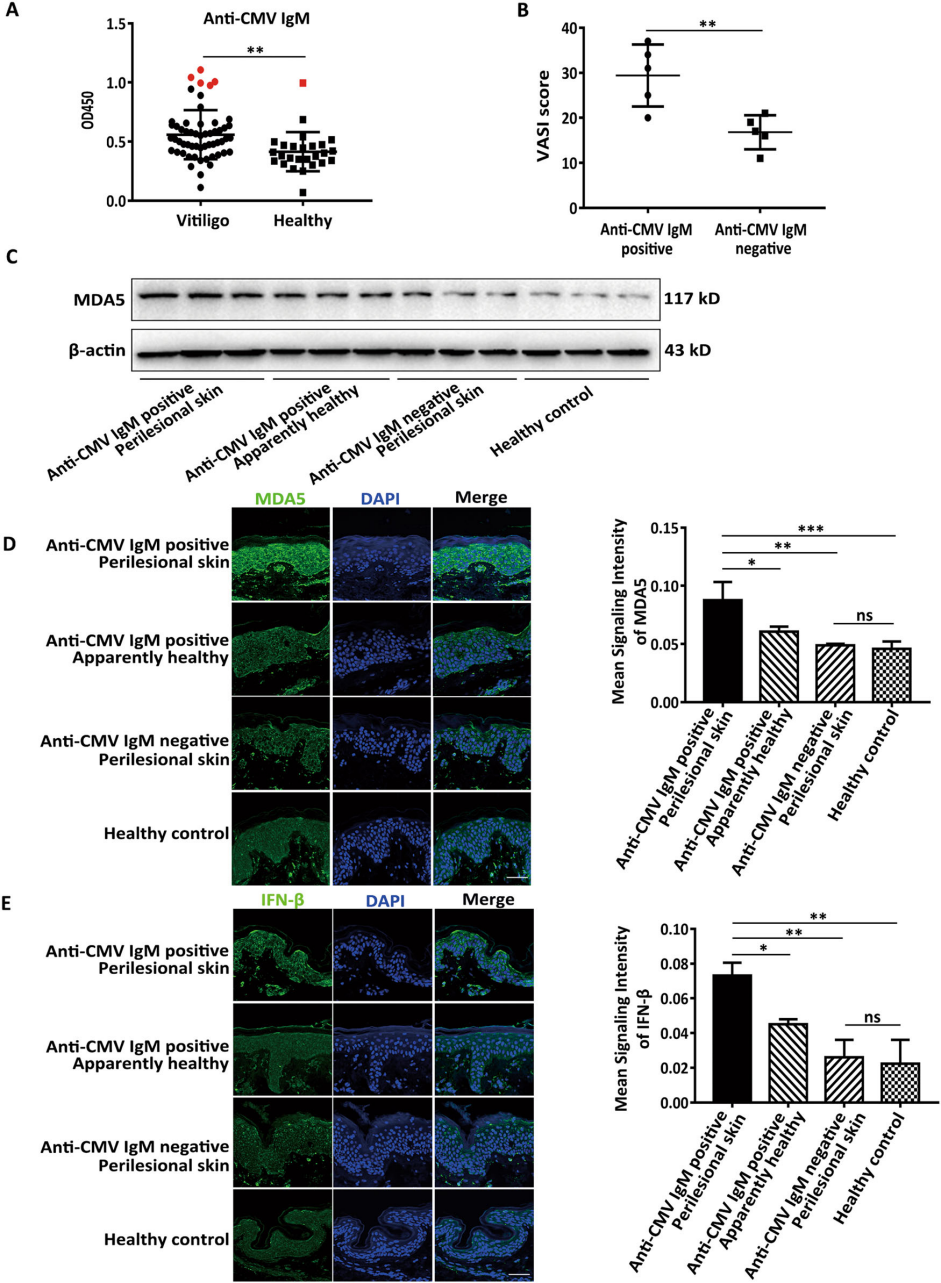
The expression of MDA5 and anti-CMV IgM is higher in some progressive vitiligo patients. **a** The anti-CMV IgM levels in 56 vitiligo patients and 26 healthy controls. The patients with the positive anti-CMV IgM were indicated in red. **b** The VASI scores of vitiligo patients with positive anti-CMV IgM or negative anti-CMV IgM. *n* = 5. **c** The Western-blot of MDA5 in the epidermis of perilesional skin of vitiligo patients with positive or negative anti-CMV IgM, in the apparently healthy epidermis of vitiligo patients with positive anti-CMV IgM and in the epidermis of healthy controls. *n* = 3. **d**, **e** The expression of MDA5 (**d**) and IFN-β (**e**) in epidermis of perilesional skin of vitiligo patients with positive or negative anti-CMV IgM, in the apparently healthy epidermis of vitiligo patients with positive anti-CMV IgM and in the epidermis of healthy controls, by immunofluorescence assay. *n* = 5, Bar = 50 μm. The data are presented as the mean ± SD across three independent experiments. ^*^P < 0.05, ^**^P < 0.01, ^***^P < 0.001. ns, not significant.

Previous study has established that the detective rate of CMV DNA in vitiligo paraffin-embedded vitiligo tissues is up to 38%^14^. To further confirm the link between virus infection and vitiligo, in the serum of progressive vitiligo we first used ELISA to detect anti-CMV IgM, symbolizing acute CMV infection^46^. We found that the positive rate of anti-CMV IgM was 8.92% in 56 progressive vitiligo patients, while that in 26 healthy controls was 3.84%, and the anti-CMV IgM levels were significantly higher in vitiligo patients than that in healthy controls (Fig. 1a). Of note, the VASI scores^47^ were higher in vitiligo patients with positive anti-CMV IgM than those with negative anti-CMV IgM (Fig. 1b). Consistently, in perilesional epidermis samples, we showed the mRNA levels of CXCL10, CXCL16 (Fig. S1A) in vitiligo patients with positive anti-CMV IgM were higher than that in patients with negative anti-CMV IgM and in healthy controls, also higher than that in the apparently healthy epidermis of vitiligo patients with positive anti-CMV IgM. In parallel, the infiltration quantity of CD8^+^ T cells in the skin tissues showed the resembled tendency (Fig. S1B). Besides, compared to healthy controls, we showed the higher expression of MDA5 (Fig. 1c, d) and IFN-β (Fig. 1e) in perilesional epidermis of vitiligo patients with positive anti-CMV IgM, while no significant upregulation in perilesional epidermis of vitiligo patients with negative anti-CMV IgM and in apparently healthy epidermis of vitiligo patients with positive anti-CMV IgM. Unlike in vitiligo, the expression of MDA5 in the lesional epidermis of psoriasis and atopic dermatitis was not apparently upregulated when compared with that of healthy controls (Fig. S2). Taken together, these data indicated that virus infection correlated with some progressive vitiligo in which MDA5 pathway might play a key role via promoting the secretion of chemokines.

### Poly(I:C) potentiates the expression of MDA5 in vitro

**Fig. 2:**
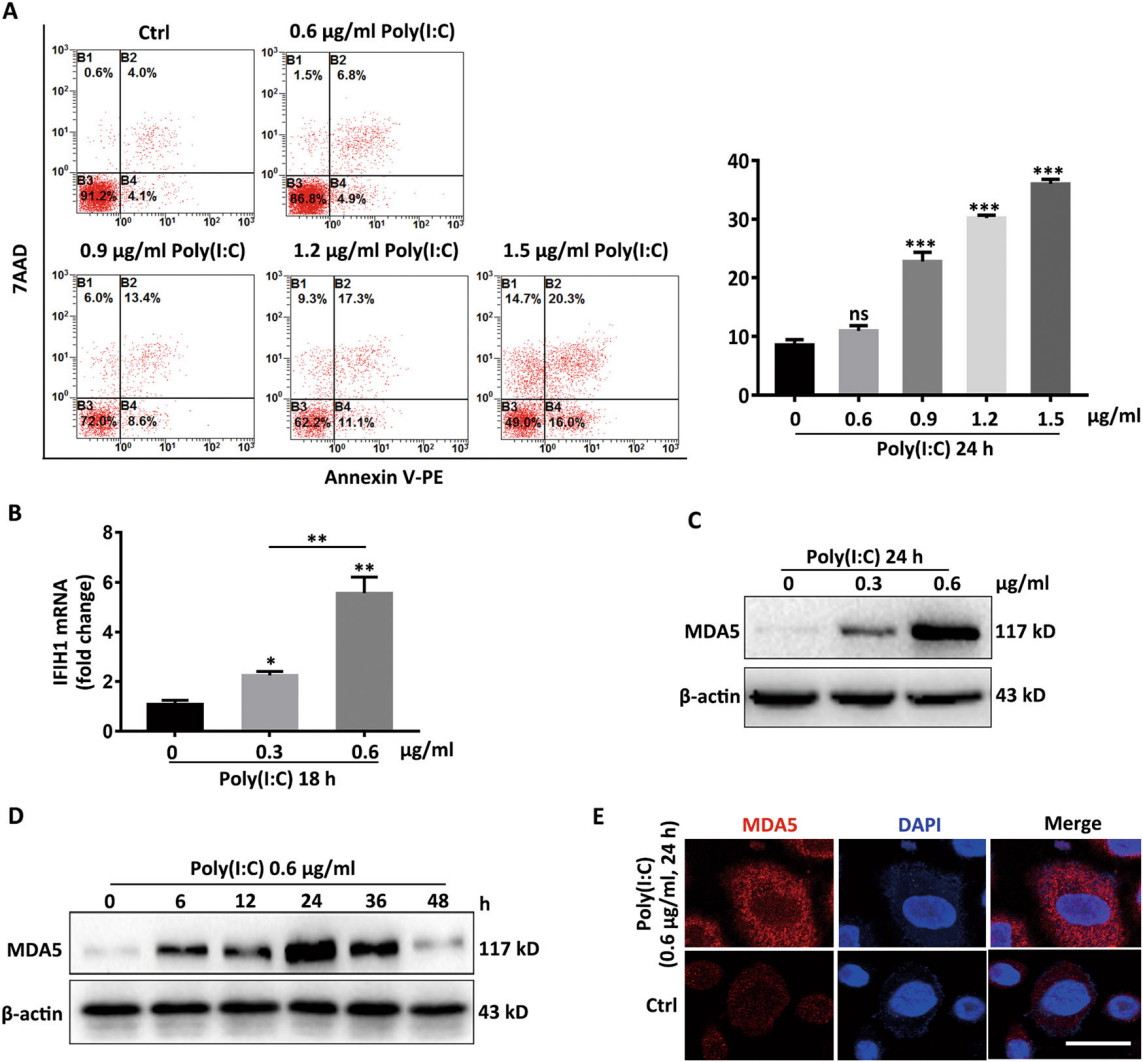
Poly(I:C) potentiates the expression of MDA5 in vitro. **a** The apoptotic rate of NHKs in response to Poly(I:C), detected by flow cytometry assay. **b**, **c** The RNA levels (**b**) and protein levels (**c**) of *IFIH1* in NHKs with Poly(I:C) concentration rose, detected by qRT-PCR assay and Western-blot respectively. **d** The expression of MDA5 in NHKs in response to 0.6 μg/ml Poly(I:C) within 48 h, determined by Western-blot assay. **e** The expression of MDA5 in NHKs stimulated by 0.6 μg Poly(I:C) for 24 h, detected by immunofluorescence assay. Bar = 10 μm. The data are representative of three independently performed experiments. ^*^P < 0.05, ^**^P < 0.01, ^***^P < 0.001. ns, not significant.

We further explored how virus invasion influenced the expression of MDA5 by applying Poly(I:C)^28,48^, a kind of virus dsRNA analog simulating the virus replication intermediate regardless of the form of nucleic acid carried by the virion^26,27^. Firstly, IC50 assay and the flow cytometry assay were performed to determine that the safety concentration of Poly(I:C) in NHKs was 0.6 μg/ml (Fig. S3 and Fig. 2a). Next, the elevated MDA5 mRNA and protein levels were observed in a Poly(I:C) concentration-dependent pattern within 0.6 μg/ml (Fig. 2b, c). In addition, the expression of MDA5 could be enhanced by Poly(I:C) in a time-dependent way within 24 h, after that the upregulated MDA5 was restored to a basic level gradually (Fig. 2d). Results from immunofluorescence assay reiterated that Poly(I:C) could upregulate the expression of MDA5 (Fig. 2e). Thus, these data manifested Poly(I:C) amplified MDA5 expression in a time- and concentration-dependent way.

### Poly(I:C) augments the secretion of CXCL10 and CXCL16

**Fig. 3:**
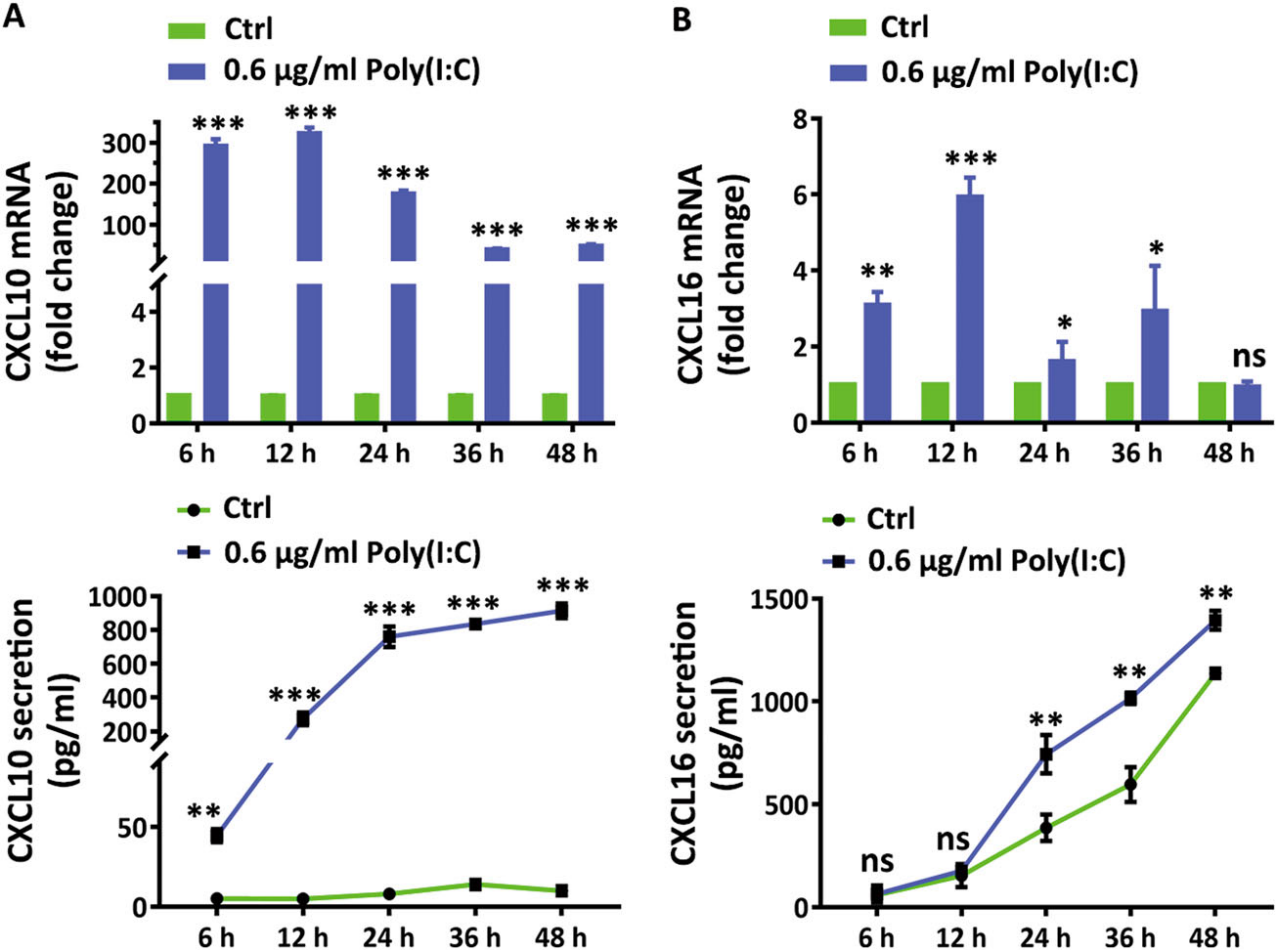
Poly(I:C) augments the expression of CXCL10 and CXCL16. **a** The levels of mRNA and secretion of CXCL10 in NHKs in response to 0.6 μg Poly(I:C) at the indicated time points. **b** The levels of mRNA and secretion of CXCL16 in NHKs in response to 0.6 μg Poly(I:C) at the indicated time points. The data are presented as the mean ± SD across three independent experiments. ^*^P < 0.05,^**^P < 0.01,^***^P < 0.001. ns, not significant.

To understand the impacts of virus invasion on keratinocytes in the pathophysiology of vitiligo, we detected the mRNA alterations of vitiligo-related cytokines and chemokines^44,45,49–53^ in response to Poly(I:C) by qRT-PCR assay. The preliminary results underlined Poly(I:C) induced drastic mRNA increments of CXCL10, CXCL16, CXCL9 and CCL20 (Fig. S4) in a concentration-dependent way. However, no significant influences of Poly(I:C) on the transcription of IL-15, CXCL12, CCL2 and CCL26 (Fig. S4) were observed. Specifically, we detected mRNA and secretion levels of CXCL10 and CXCL16 by qRT-PCR and ELISA respectively, and found that Poly(I:C) potentiated the mRNA and secretion levels of CXCL10 and CXCL16 within 48 h (Fig. 3a, b). These data indicated that Poly(I:C) dictated keratinocytes to generate redundant CXCL10 and CXCL16.

### The secretion of CXCL10 and CXCL16 is orchestrated by the combination of MDA5 and MAVS

**Fig. 4:**
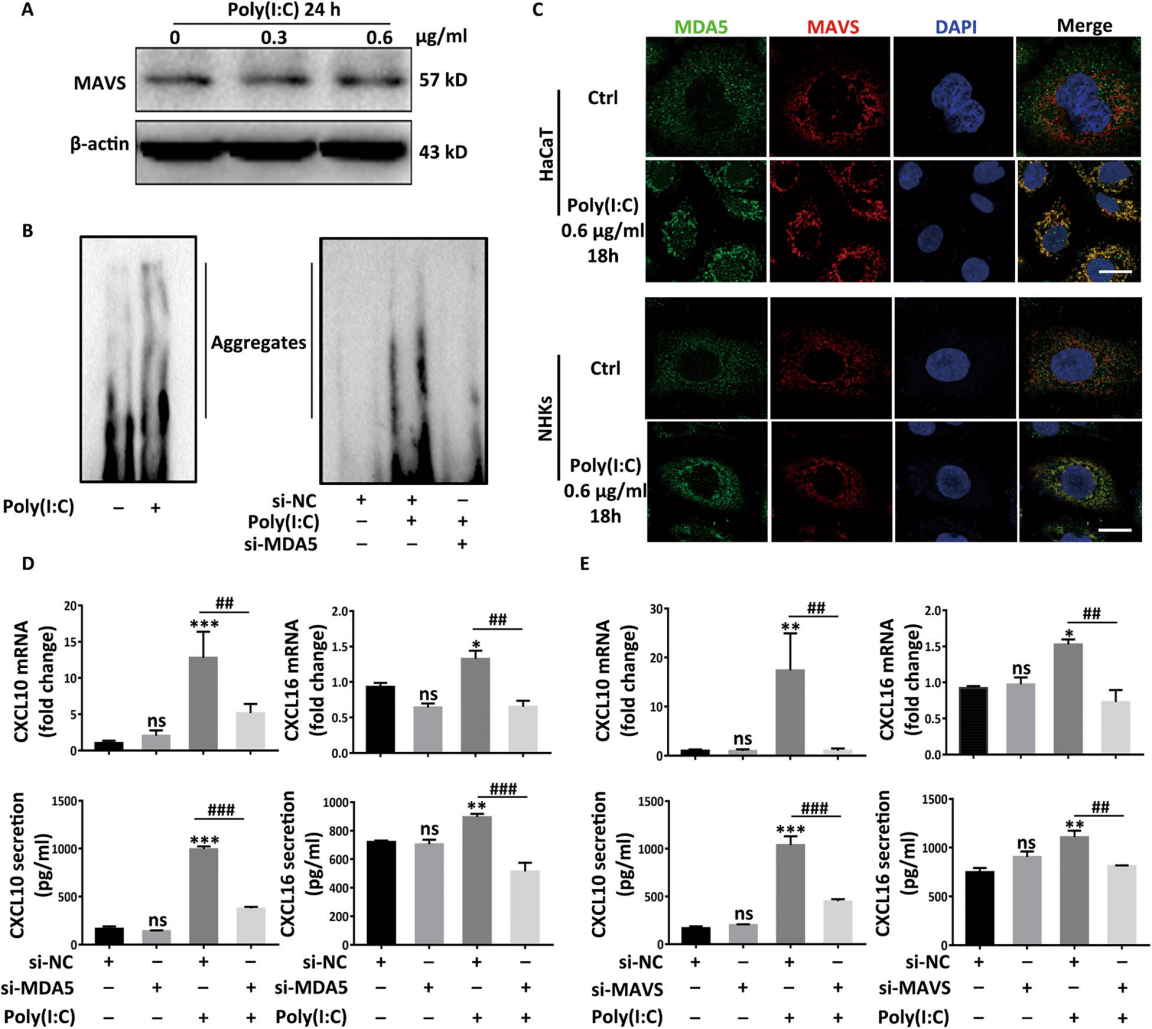
The secretion of CXCL10 and CXCL16 is orchestrated by the combination of MDA5 and MAVS. **a** The Western-blot of MAVS in NHKs in response to Poly(I:C). **b** The formation of MAVS aggregates with the treatment of Poly(I:C) for 18 h in NHKs, or interfering MDA5 by using MDA5 siRNA (si-MDA5) prior to 18 h Poly(I:C) treatment in HaCaT cells, detected by SDD-AGE assay. **c** The colocalization of MDA5 and MAVS in NHKs and HaCaT cells, detected by immunofluorescence assay. Bar = 10 μM. **d**, **e** The mRNA and secretion levels of CXCL10 and CXCL16 when interfering MDA5 (**d**) or interfering MAVS by using MAVS siRNA (si-MAVS) (**e**) prior to Poly(I:C) treatment in HaCaT cells. The data are presented as the mean ± SD across three independently performed experiments. ^*^P < 0.05, ^**^P < 0.01, ^***^P < 0.001, ^#^*P* < 0.05, ^##^*P* < 0.01, ^###^*P* < 0.001. ns, not significant.

Unlike MDA5 functioning potently in an expression-upregulation way, MAVS mainly depends on the formation of aggregates to exert its functions^36^. Consistent with this, we found that the contents of MAVS manifested no obvious change (Fig. 4a) while that of MDA5 significantly mounted in NHKs (Fig. 2c). Alongside, SDD-AGE exhibited formation of MAVS aggregates^38^ following Poly(I:C) stimulation (Fig. 4b) for 18 h in NHKs. Of note, conspicuous diminution of MAVS aggregates emerged when interfering MDA5 with siRNA (si-MDA5) (Fig. 4b) in HaCaT cells. Furthermore, the immunofluorescence assay suggested that MDA5, randomly scattering in cytoplasm in stationary phase, colocalized with MAVS swiftly and convened around cell nucleus in response to Poly(I:C) (Fig. 4c). Sequentially, we knocked down MDA5 by using siRNA (Fig. S5A) and found that interfering MDA5 significantly lessened CXCL10 and CXCL16 (Fig. 4d) at both mRNA and secretion levels. Also, interfering MAVS by using siRNA (si-MAVS) had the comparable results (Fig. S5B and Fig. 4e). These data demonstrated that the expression of CXCL10 and CXCL16 is orchestrated by the combination of MDA5 and MAVS with the Poly(I:C) stimulation.

### NF-κB and IRF3 mediates the secretion of CXCL10 and CXCL16

**Fig. 5:**
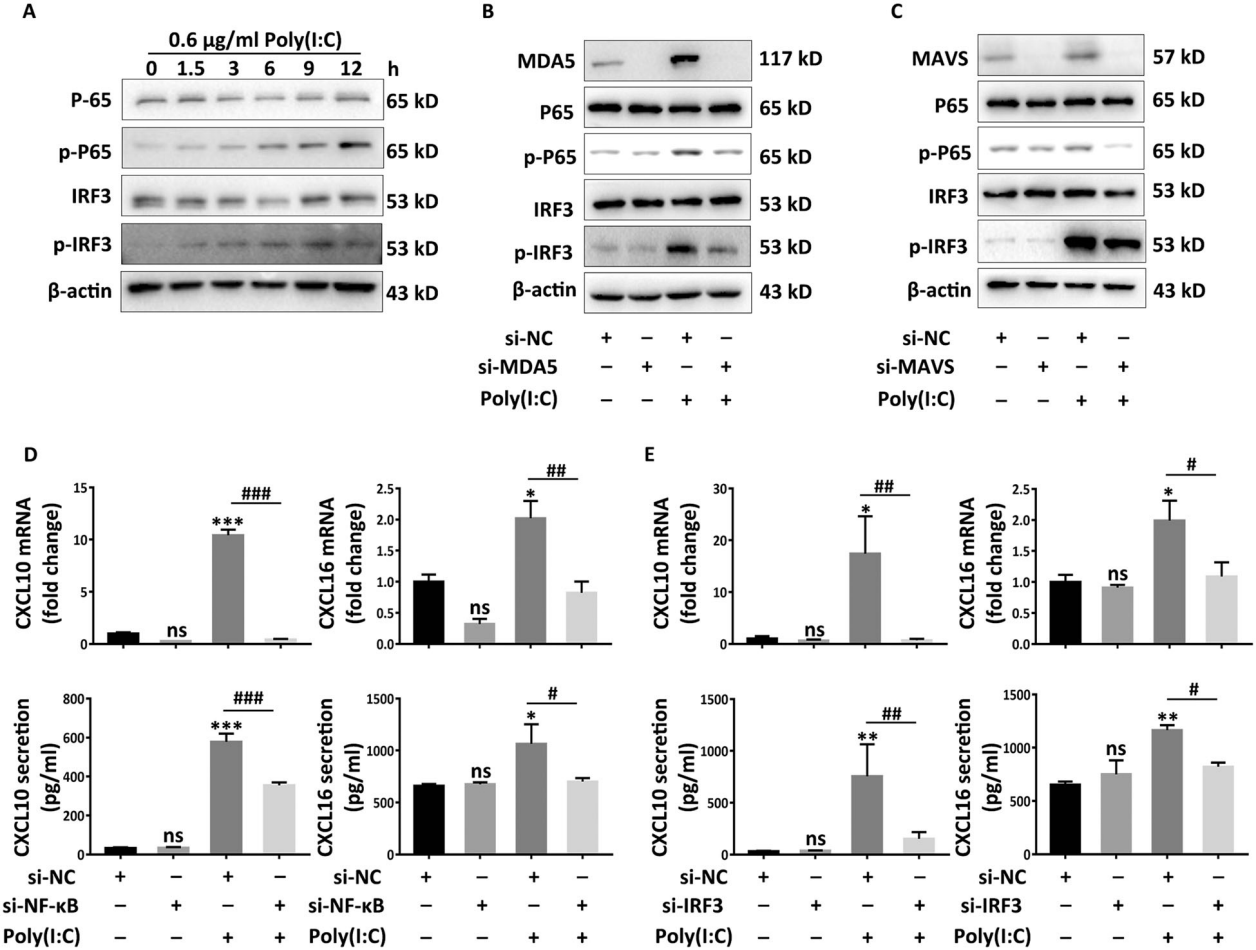
NF-κB and IRF3 is decisive for secretion of CXCL10 and CXCL16. **a** The Western-blots of total P-65, phosphor-P65, total IRF3 and phosphor-IRF3 in NHKs in the indicated time in response to Poly(I:C). **b**, **c** The Western-blots of total P-65, phosphor-P65, total IRF3 and phosphor-IRF3 when interfering MDA5 (si-MDA5) (**b**) or interfering MAVS (si-MAVS) (**c**) in HaCaT cells prior to 24 h 0.6 μg/ml Poly(I:C) treatment. **d**, **e** The mRNA and secretion levels of CXCL10 and CXCL16 when interfering NF-κB by using NF-κB siRNA (si-NF-κB) (**d**) or interfering IRF3 by using IRF3 siRNA (si-IRF3) (**e**) for 24 h prior to 0.6 μg/ml Poly(I:C) stimulation for 24 h in HaCaT cells. The data are presented as the mean ± SD across three independently performed experiments. ^*^P < 0.05, ^**^P < 0.01, ^***^P < 0.001, ^#^*P* < 0.05, ^##^*P* < 0.01, ^###^*P* < 0.001. ns, not significant.

As for IFN-β production, previous studies have defined critical roles of NF-κB and IRF3 as signal transducer in the downstream of MAVS^54^, which provides a template for understanding the mechanisms mediating the secretion of CXCL10 and CXCL16. With Poly(I:C) treatment, Western-blot results manifested that the expression of phospho-NF-κB P65 and phospho-IRF3 apparently increased in a time-dependent manner (Fig. 5a), which were diminished with the pretreatment of siRNA targeting MDA5 in HaCaT cells (Fig. 5b). Similarly, impeding MAVS also repressed the activation of NF-κB and IRF3 (Fig. 5c). Next, we silenced NF-κB and IRF3 by using corresponding siRNA (si-NF-κB and si-IRF3) to investigate the alterations of chemokines in mRNA and secretion levels in response to Poly(I:C) (Fig. S5C-5D). Results showed that interfering either NF-κB or IRF3 drastically dampened expression of CXCL10 and CXCL16 at both mRNA and secretion levels (Fig. 5d, e). These data underpinned critical roles of NF-κB and IRF3 in CXCL10 and CXCL16 secretion in the downstream of MAVS in keratinocytes.

### MDA5-mediated IFN-β is central to the secretion of CXCL10 while CXCL16 is directly regulated by IRF3

**Fig. 6:**
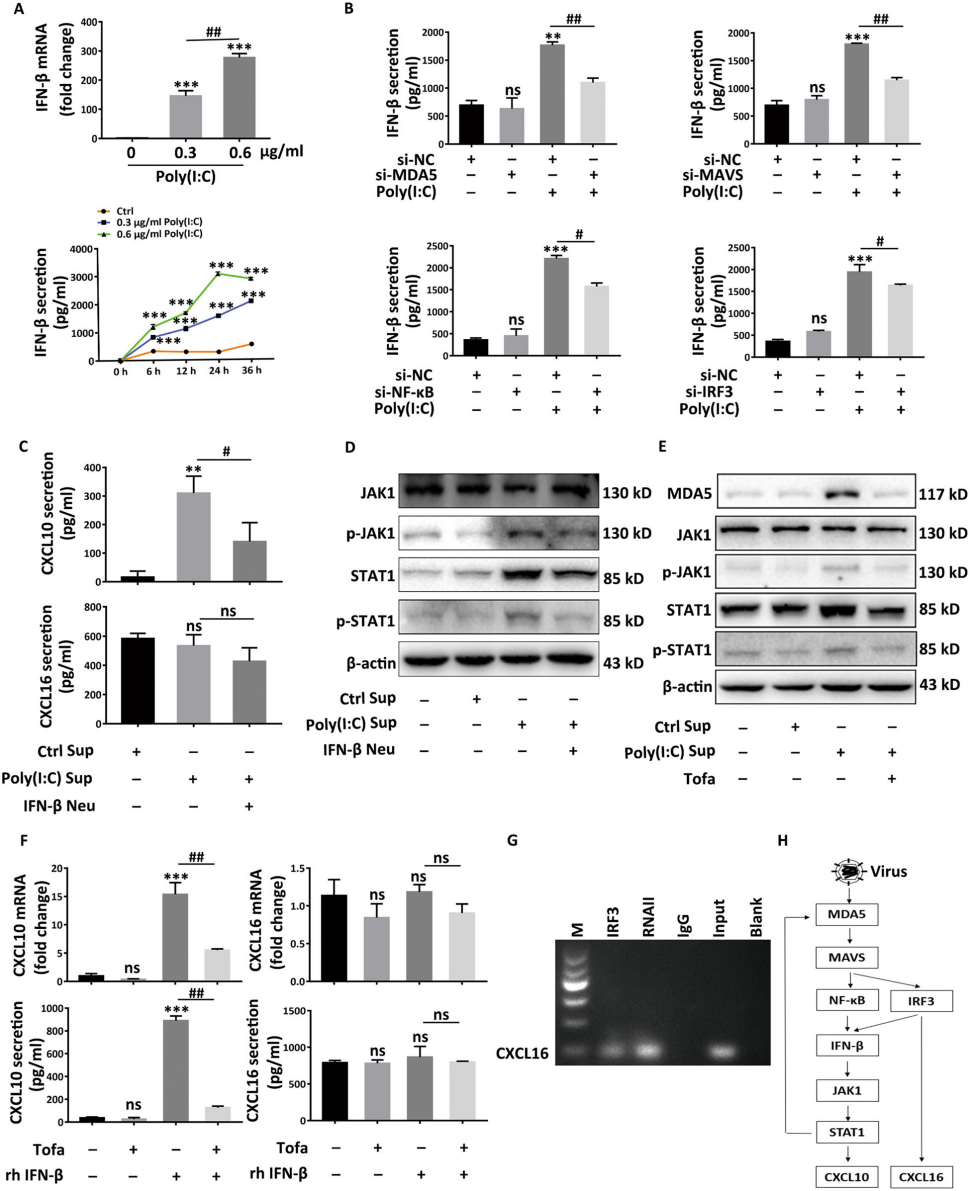
MDA5-mediated IFN-β is central to the secretion of CXCL10 while CXCL16 is directly regulated by IRF3. **a** The mRNA and secretion levels of IFN-β in HaCaT cells in response to Poly(I:C). **b** The secretion levels of IFN-β when interfering any of MDA5, MAVS, NF-kB, IRF3 prior to 24 h treatment of Poly(I:C) in HaCaT cells. **c** The secretion levels of CXCL10 and CXCL16 when treated with Poly(I:C)-pretreated supernatant (Sup) with or without neutralizing the IFN-β by using the IFN-β neutralizing antibody (Neu), detected by ELISA assay. **d**, **e** The expression of total JAK, phosphor-JAK, total STAT1, phosphor-STAT1 and MDA5 in HaCaT cells exposed to the Poly(I:C)-pretreated supernatant (Sup), and simultaneously with or without neutralizing IFN-β (Neu) (**d**) or blocking JAK1-STAT1 pathway with the pretreatment of Tofacitinib (Tofa) (**e**). **f** The mRNA and secretion levels of CXCL10 and CXCL16 in HaCaT cells stimulated by recombined human IFN-β (rh IFN-β) or pretreated with Tofacitinib (Tofa), detected by ELISA. **g** Chromatin immunoprecipitation assay (ChIP) of IRF3 to CXCL16 promoter after the HaCaT cells were treated by Poly(I:C) for 18 h. **h** The diagram of the pathophysiological process in keratinocytes under the virus invasion. Data are presented as the mean ± SD across three independently performed experiments. ^**^P < 0.01, ^***^P < 0.001, ^#^*P* < 0.05, ^##^*P* < 0.01. ns, not significant.

In HaCaT cells, we further determined whether the secretion of CXCL10 and CXCL16 is mediated by IFN-β in response to Poly(I:C) stimulation. We proved Poly(I:C) potentiated the transcription of IFN-β in a dose-dependent way as shown by qRT-PCR assay and promoted the secretion of IFN-β in a time and concentration-dependent manner (Fig. 6a). Interfering any of MDA5, MAVS, NF-κB, and IRF3 could potently attenuate IFN-β secretion (Fig. 6b). Then, after 24 h Poly(I:C) treatment in HaCaT cells, we collected the supernatant (Sup) to treat HaCaT cells for 24 h. After subtracting the basic CXCL10 and CXCL16, we found the supernatant could elevate the secretion of CXCL10 but not CXCL16. While neutralizing IFN-β using IFN-β neutralizing antibody (Neu) in supernatant disappeared the effect of promoting CXCL10 (Fig. 6c). Previous study verified that JAK1-STAT1 signaling functions in the downstream of IFN-β^55^. Consistently, we found the Poly(I:C)-stimulated supernatant could apparently promote the expression of MDA5 and activate JAK1-STAT1 signaling pathway featuring the upregulation of phospho-JAK1 and phospho-STAT1 (Fig. 6d, e). Either using IFN-β neutralizing antibody (Fig. 6d) in the supernatant or pretreating HaCaT cells with the inhibitor of JAK1, Tofacitinib (Tofa) (Fig. 6e), impeded the activation of JAK1-STAT1 signaling pathway, which indicated JAK1-STAT1 signaling pathway functioned in the downstream of IFN-β and this pathway could trigger the expression of MDA5 to form a positive feedback loop (Fig. 6e). What’s more, we discovered that recombined human IFN-β (rh IFN-β) indeed promoted the CXCL10 rather than CXCL16 at both mRNA and secretion levels (Fig. 6f). Consistently, pretreatment with Tofacitinib nullified the elevated secretion of CXCL10 elicited by rh IFN-β (Fig. 6f). To explore the mechanism underlying the regulation of the CXCL16, we screened the JASPAR database (http://jaspar.genereg.net) and found the -2000 to +1 region of the CXCL16 promoter has 2 predicted binding sites for IRF3 (Fig. S6). The ChIP results also validated that IRF3 could bind directly to the promotor of CXCL16 and further promoted the transcription of CXCL16 under virus invasion (Fig. 6g). These data indicated MDA5-mediated IFN-β is central to the secretion of CXCL10 in the JAK1-STAT1-dependent fashion, while CXCL16 is directly regulated by IRF3 via binding to the promotor of CXCL16 (Fig. 6h).

## Discussion

In the present study, we initially manifested that the expression of MDA5 and anti-CMV IgM was upregulated in some progressive vitiligo. Then in vitro assays verified that virus infection, simulated by Poly(I:C), could promote the secretion of CXCL10 and CXCL16. Mechanistically, we demonstrated that IFN-β mediated by the MDA5-MAVS-NF-κB/IRF3 signaling pathway orchestrated the secretion of CXCL10 via the JAK1-STAT1 pathway and MDA5-meidiated IRF3 transcriptionally induced the production of CXCL16 in keratinocytes under virus invasion. In short, we proved that targeting MDA5 signaling pathway has the potent therapeutic potential in retarding the aggravation of vitiligo under virus invasion.

Increasing evidences have indicated the correlation between virus infection and autoimmune diseases such as Coxsackie virus and type 1 diabetes^6^, and the potential mechanisms embody adjuvant initiative effect of autoimmune reaction, molecule mimicry, bystander activation and epitope spreading^56^. Vitiligo, as an autoimmunity-involved disease, is thought to correlate with virus infection^14–16,19–22^. Recently, *IFIH1*, encoding MDA5 which is a virus-associated pattern recognition receptor (PRR), has been identified as a susceptible gene for vitiligo^3^. Herein, we found the positive rate and levels of anti-CMV IgM in the serum of progressive vitiligo patients were significantly higher than that in healthy controls. In addition, our data showed the expression of MDA5 and IFN-β was enhanced in keratinocytes of vitiligo patients with positive anti-CMV IgM. As the sporadic evidences indicated, although MDA5 could also be activated by some self-derived dsRNA^57,58^ in vivo, the human evolves many crucial mechanisms to prevent the activation of *IFIH1* via inhibiting the production of endogenous recognizable RNA^57–60^. More importantly, given that the higher expression of MDA5 and IFN-β is along with the positive anti-CMV IgM in some progressive vitiligo patients, we attribute the higher expression of MDA5 in the vitiligo patients with positive anti-CMV IgM to virus infection. All these above strengthen that virus infection is involved in some progressive vitiligo pathogenesis and indicate that MDA5 plays a key role in vitiligo under virus invasion.

MDA5, as a cytosolic virus sensor, contributes significantly to the elimination of virus in end-organs not only through the induction of antiviral interferons and other pro-inflammatory cytokines, but also by facilitating cell death^5^. Meanwhile, the inappropriate gain-of-function of MDA5 could efficiently eradicate virus, but at the cost of increasing incidence to suffer autoimmune disease such as type-1 diabetes (T1D)^61^ and systemic lupus erythematosus (SLE)^62^. This argues against that the persistent viral infection *per se* induces autoimmunity and indicates that MDA5-mediated aberrant skin immunity functions prominently^6,63^. Herein, we highlighted the impacts of MDA5 on the dysfunctional local immune microenvironment, particularly on the secretion promotion of CXCL10 and CXCL16 under virus invasion in keratinocytes. Previous studies have in succession clarified that CXCL10^43^ and CXCL16^45^ play key roles in the trafficking of melanocyte-specific autoreactive CD8^+^ T cells from periphery blood to skin, which contributed significantly to melanocytes death in vitiligo. Specifically, the in vivo assay conducted in a mouse vitiligo model verified that, mice received *Cxcr3*^*-/-*^ T cells develop minimal depigmentation, as do mice lacking *Cxcl10* or treated with CXCL10-neutralizing antibody. In addition, CXCL10 neutralization treatment in vitiligo mice with the established and widespread depigmentation induces repigmentation^43^. Besides, functional study unveiled that the IFN-γ-JAK-STAT signaling axis is required for CXCL10 secretion in epidermis of vitiligo^44^ and blockade of this pathway using tofacitinib or ruxolitinib which are all JAK inhibitor rapidly induces the repigmentation of vitiligo^64,65^. With respect to the important roles of CXCL16 in vitiligo, we previously demonstrated that compared with nonlesional and healthy control skin, more CXCR6^+^ CD8^+^ T cells are located at the basal epidermis and dermis of perilesions from vitiligo patients, which is consistent with the higher content of CXCL16 in epidermis. Besides, in vitro assay verified that neutralizing CXCL16 in the supernatant of H_2_O_2_-treated primary keratinocytes significantly decreases the migration of CD8^+^ T cells sorted from vitiligo patients^45^. All these above further underscore the significance of MDA5-mediated chemokines in the pathogenesis of vitiligo. In addition, Previous studies have proved that IFN-γ signature^44,49^ and oxidative stress^45^ play key parts in the secretion of chemokines in keratinocytes. Our data further complement the specific mechanisms of chemokines secretion from keratinocytes during the process of melanocytes death and extend the underlying mechanism by which virus infection engages in autoimmune disease.

Notably, previous studies also emphasized the roles of IFN-β in reinforcing and bridging the innate and adaptive immunology such as promoting dendritic cells (DCs) maturation, skewing of TH1 polarization and reactivation of CTLs (cytotoxic T lymphocyte)^41,66^. Besides, MDA5 belongs to the RIG-I-like receptor family (RLR family) which could initiate the production of pro-inflammatory factors including IL-6, IL-8, IL-23, and TNF-α^31,67,68^. Hence, whether other functions of MDA5 participate in the pathogenesis of vitiligo in response to virus invasion is worthy to be explored.

Mechanistically, we demonstrated that MDA5 exerted its function by inducing the formation of MAVS aggregates after the colocalization of MDA5 and MAVS in keratinocytes. This result parallels the canonic MDA5 signaling pathway that, MDA5 binds to viral dsRNA through the C-terminal RD domain and sequentially the exposed N-terminal CARD domains interact with MAVS which rapidly forms the prion-like aggregates^36^. What’s more, our data validated that the Poly(I:C)-stimulated keratinocytes supernatant had no impacts on the CXCL16 secretion and IRF3 could directly bind to the promotor of CXCL16 in response to virus. Combined with our previous finding that NF-κB P65 could directly mediate the transcription of CXCL16 in keratinocytes through directly binding to the promotor of CXCL16^45^, we draw a conclusion that CXCL16 is mainly elaborated by IRF3 and NF-κB in the downstream of MAVS under virus infection. As for CXCL10, we exhibited that it was orchestrated to a large extent by IFN-β which was induced by the MDA5 canonic pathway. Notably, another study underscored that Hantaan virus, the pathogen of hemorrhagic fever with renal syndrome (HFRS), promotes the secretion of CXCL10 by facilitating NF-κB and IRF7 to directly bind to the promotor of CXCL10 in the downstream of MDA5^69^. This supports that NF-κB might mediate the secretion of CXCL10 not merely through IFN-β but also via prompting the transcription of CXCL10 directly. Moreover, our data exhibited that IFN-β could significantly prompt the expression of MDA5 via the JAK1-STAT1 signaling pathway, thus forming a positive feedback loop to aggravate this pathogenesis.

The present study deciphers that MDA5 could exacerbate vitiligo by inducing the secretion of chemokines in keratinocytes under virus invasion. Even so, the specific causal relationship are still difficult to solidly identify due to the complex interaction between virus infection and vitiligo such as the “hit and run” hypothesis of virus^6,56^. Hence, identifying the pathological evidence of specific virus or the solid causative link with virus infection in vitiligo warrants further investigation. In addition, based on our present data, in vivo assay should be further performed to explore the remarkable contribution of virus invasion in the exacerbation of vitiligo via the MDA5-chemokines pathway. Aiming at individualized treatment, we think our research could provide the evidence to the MDA5-targeting therapy in vitiligo under virus invasion.

## Supplementary information

Supplementary Materials

Fig. S1

Fig. S2

Fig. S3

Fig. S4

Fig. S5

Fig. S6
