# Supplementary material for: Intracellular virus sensor MDA5 exacerbates vitiligo by inducing the secretion of chemokines in keratinocytes under virus invasion
Source: Cell Death Dis. 2020 Jun 12;11(6):453. doi: 10.1038/s41419-020-2665-z (PMC7293308; doi:10.1038/s41419-020-2665-z)
Supplement: Supplementary file 1 — Supplementary Materials [file 41419_2020_2665_MOESM1_ESM.doc]

**Supplementary methods**

[**Cytomegalovirus**](javascript:;) **IgM detection**

Serum samples from 56 vitiligo patients and 26 healthy controls were equilibrated to the room temperature from stored deep-frozen (-20 to -80 °C) prior to use. Next, all samples were diluted 1:100 with IgM Sample Diluent and were added into appropriate wells. Different from the regular ELISA kit which needs a standard curse to quantify the content of samples, there is no standard curve in this ELISA assay. Instead, this ELISA kit consists of all controls (Cytomegalovirus IgM Positive, Cytomegalovirus IgM Negative and Cytomegalovirus IgM Cut-off) to determine test results. After incubating for 1 hour at 37 °C, aspirating the content of every wells and washing well with 300 μL 1×Washing solution for three times. Then 100 μL Cytomegalovirus anti-IgM HRP Conjugate was added into each well for 30 minutes at room temperature without direct sunlight. After washing for 3 times, adding 100 μL TMB Substrate Solution into all wells for 15 minutes in dark. Last, adding 100 μL Stop Solution into all wells. Within 30 minutes after adding the Stop Solution, the absorbance of the specimens was measured at 450 nm. At last, calculating the mean background subtracted absorbances for each sample and compare to mean Cut-off control value. Samples are considered to give a positive signal if the absorbance value is greater than 10% over the mean cut-off value. While that the absorbance values of two independent experiments were all less than 10% above or below the mean cut-off control value was considered as negative.

**Supplementary Figure Legends**

**Figure S1.** A. The mRNA levels of CXCL10 and CXCL16 in the perilesional epidermis and apparently healthy epidermis of vitiligo patients with positive anti-CMV IgM or in the perilesional epidermis of patients with negative anti-CMV IgM, or in the epidermis of healthy controls. B. The infiltration quantity of CD8^+^ T cells in the perilesional skin and apparently healthy skin of vitiligo patients with positive anti-CMV IgM or in the perilesional skin of patients with negative anti-CMV IgM, or in the skin of healthy controls. Bar = 100 μm. The data represents the results of three independent experiments and are shown by means ± SD. ^*^P＜0.05, ^**^*P*＜0.01, ^***^*P*＜0.001. ns, not significant.

**Figure S2.** The expression of MDA5 in the epidermis of atopic dermatitis and psoriasis, determined by Western-blot. The data represents the results of three independent experiments and are shown by means ± SD. ns, not significant.

**Figure S3.** The IC50 value of Poly(I:C) in NHKs is 1.4 μg/ml, determined by CCK8 assay. The data represents the results of three independent experiments.

**Figure S4.** Poly(I:C) could promote the mRNA increment of vitiligo-related cytokines in NHKs. The mRNA levels of CXCL10, CXCL16, CCL20, CXCL9, IL-15, CCL26, CXCL12, CCL2 in NHKs treated with Poly(I:C) for 18 hours, detected by qRT-PCR assay. The data represents the results of three independent experiments and are shown by means ± SD. ^#^*P*＜0.05，^##^*P*＜0.01，^**^*P*＜0.01, ^***^*P*＜0.001. ns, not significant.

**Figure S5.** The determination of siRNA interference efficiency. The interference efficiency of small interference RNA (siRNA) targeting MDA5 **(A)**, MAVS **(B)**, NF-κB P65 **(C)** and IRF3 **(D)** respectively, determined by Western-blot assay. The data represents the results of three independent experiments.

**Figure S6.** Potential binding sites of the transcription factor IRF3 in the -2000 to -1 region of the CXCL16 promoter predicted by using the JASPAR database shown in red and overlined.

**Supplementary Tables**

**Supplementary Table 1 The numbers of subjects in each assay.**

| Category | NO. | VASI score | Immuno-  fluorescence for MDA5 and IFN-β | qRT-PCR for  CXCL10 and CXCL16 | Western-blot for MDA5 |
| --- | --- | --- | --- | --- | --- |
| Anti-CMV IgM  positive vitiligo | 54054 | yes | yes | yes | yes |
|  | 72815 | yes | yes | yes | yes |
|  | 53708 | yes | yes | yes | yes |
|  | 49422 | yes | yes | yes | no |
|  | 53713 | yes | yes | yes | no |
| Anti-CMV IgM  negative vitiligo | 53478 | yes | yes | yes | yes |
|  | 47283 | yes | yes | yes | yes |
|  | 49546 | yes | yes | yes | yes |
|  | 49554 | yes | yes | yes | no |
|  | 67453 | yes | yes | yes | no |
| Healthy controls | 1 | no | yes | yes | yes |
|  | 2 | no | yes | yes | yes |
|  | 3 | no | yes | yes | yes |
|  | 4 | no | yes | yes | no |
|  | 5 | no | yes | yes | no |

**Supplementary Table 2 The primary antibodies used in the Western-blot assays.**

| Primary Antibody | Dilution | Corporation | Country | Catalog |
| --- | --- | --- | --- | --- |
| rabbit monoclonal anti-MDA5 | 1:1000 | Abcam | USA | ab126630 |
| mouse monoclonal anti-MAVS | 1:500 | Abcam | USA | ab220170 |
| mouse monoclonal anti-NF-κB P65 | 1:1000 | CST | USA | 6956 |
| rabbit monoclonal anti-NF-κB p-P65 | 1:1000 | CST | USA | 3033 |
| rabbit monoclonal anti-IRF3 | 1:1000 | CST | USA | 11904 |
| rabbit monoclonal anti-p-IRF3 | 1:1000 | CST | USA | 29047 |
| rabbit monoclonal anti-JAK1 | 1:1000 | CST | USA | 29261 |
| rabbit monoclonal anti-p-JAK1 | 1:1000 | CST | USA | 74129 |
| rabbit monoclonal anti-STAT1 | 1:1000 | CST | USA | 14994 |
| rabbit monoclonal anti-p-STAT1 | 1:1000 | CST | USA | 9167 |

**Supplementary Table 3 The primer sequences used in the qRT-PCR assays.**

| Genes | Forward | Reverse |
| --- | --- | --- |
| *IFIH1* | TGGTGGACAAGCTTCTAGTTAG | GTTTCCTGTTTGACGAAGAACA |
| *CXCL10* | CTCTCTCTAGAACTGTACGTCG | ATTCAGACATCTCTTCTCACCC |
| *IFNβ* | TGGCTGGAATGAGACTATTGTT | GGTAATGCAGAATCCTCCCATA |
| *CXCL12* | CCAACGTCAAGCATCTCAAAAT | CACACTTGTCTGTTGTTGTTC |
| *CCL2* | AGAATCACCAGCAGCAAGTGTCC | TTGCTTGTCCAGGTGGTCCATG |
| *CXCL16* | CGTCACTGGAAGTTGTTATTGT | GTATAGACACCGATGGTAAGCT |
| *CXCL9* | AAGACCTTAAACAATTTGCCCC | TGCTGAATCTGGGTTTAGACAT |
| *IL-15* | TCCAGTGCTACTTGTGTTTACT | CTGAAACAGCCCAAAATGAAGA |
| *CCL26* | GGAGTGACATATCCAAGACCTG | CTTGGATGGGTACAGACTTTCT |
| *CCL20* | ACTTTGACTGCTGTCTTGGATA | GACCCAAGTCTGTTTTGGATTT |
