# Supplementary figures and images for: Intracellular virus sensor MDA5 exacerbates vitiligo by inducing the secretion of chemokines in keratinocytes under virus invasion
Source: Cell Death Dis. 2020 Jun 12;11(6):453. doi: 10.1038/s41419-020-2665-z (PMC7293308; doi:10.1038/s41419-020-2665-z)

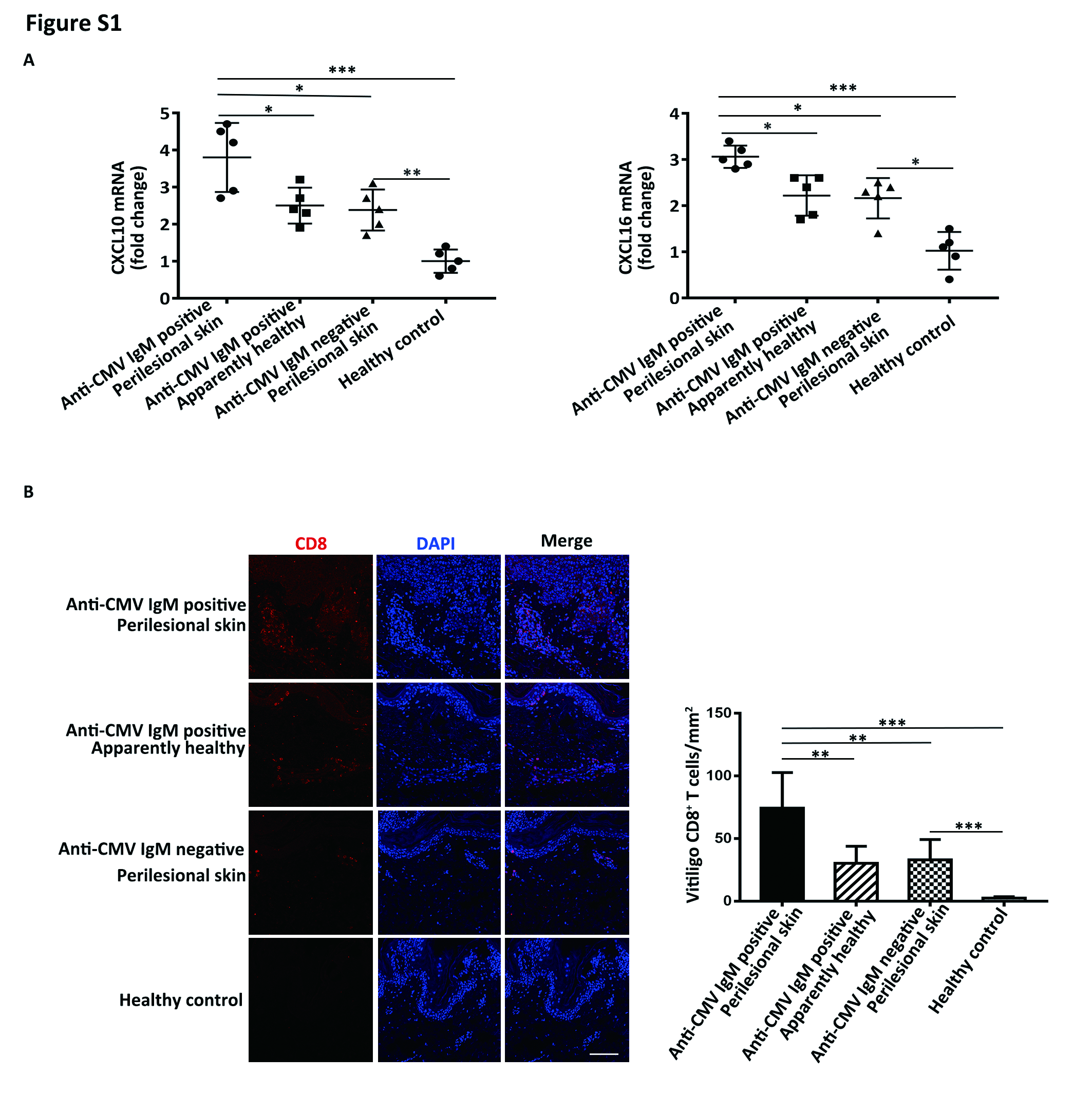

Supplement: Supplementary file 2 — Fig. S1 [file 41419_2020_2665_MOESM2_ESM.tif]

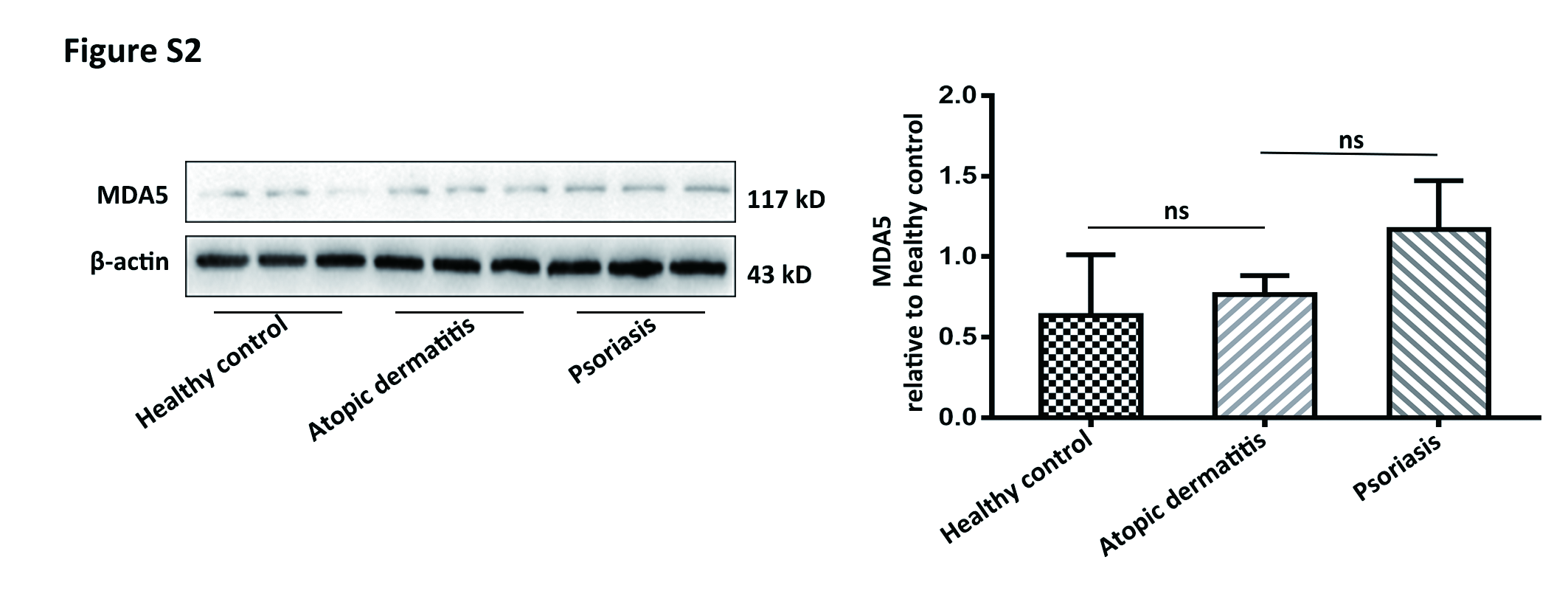

Supplement: Supplementary file 3 — Fig. S2 [file 41419_2020_2665_MOESM3_ESM.tif]

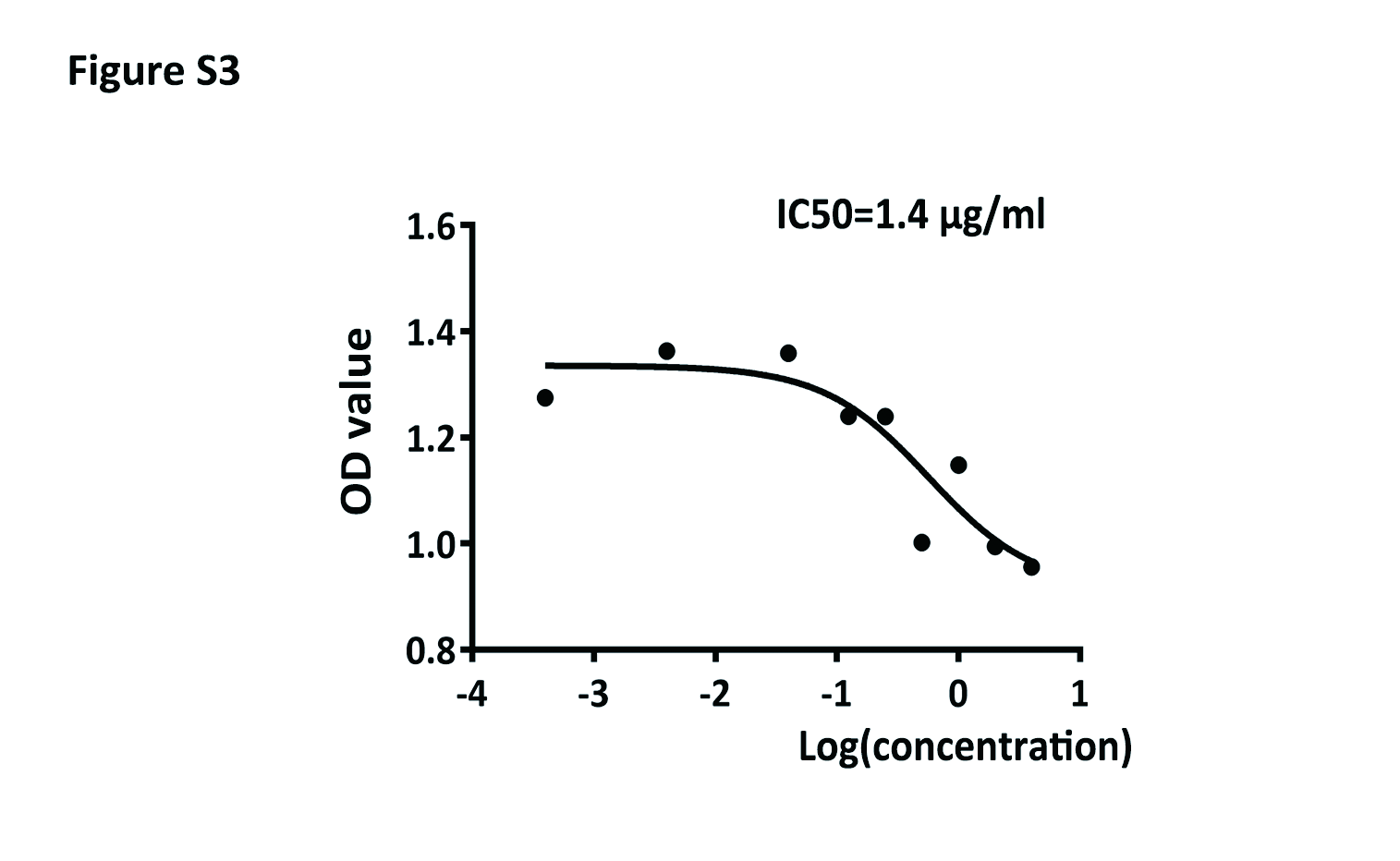

Supplement: Supplementary file 4 — Fig. S3 [file 41419_2020_2665_MOESM4_ESM.tif]

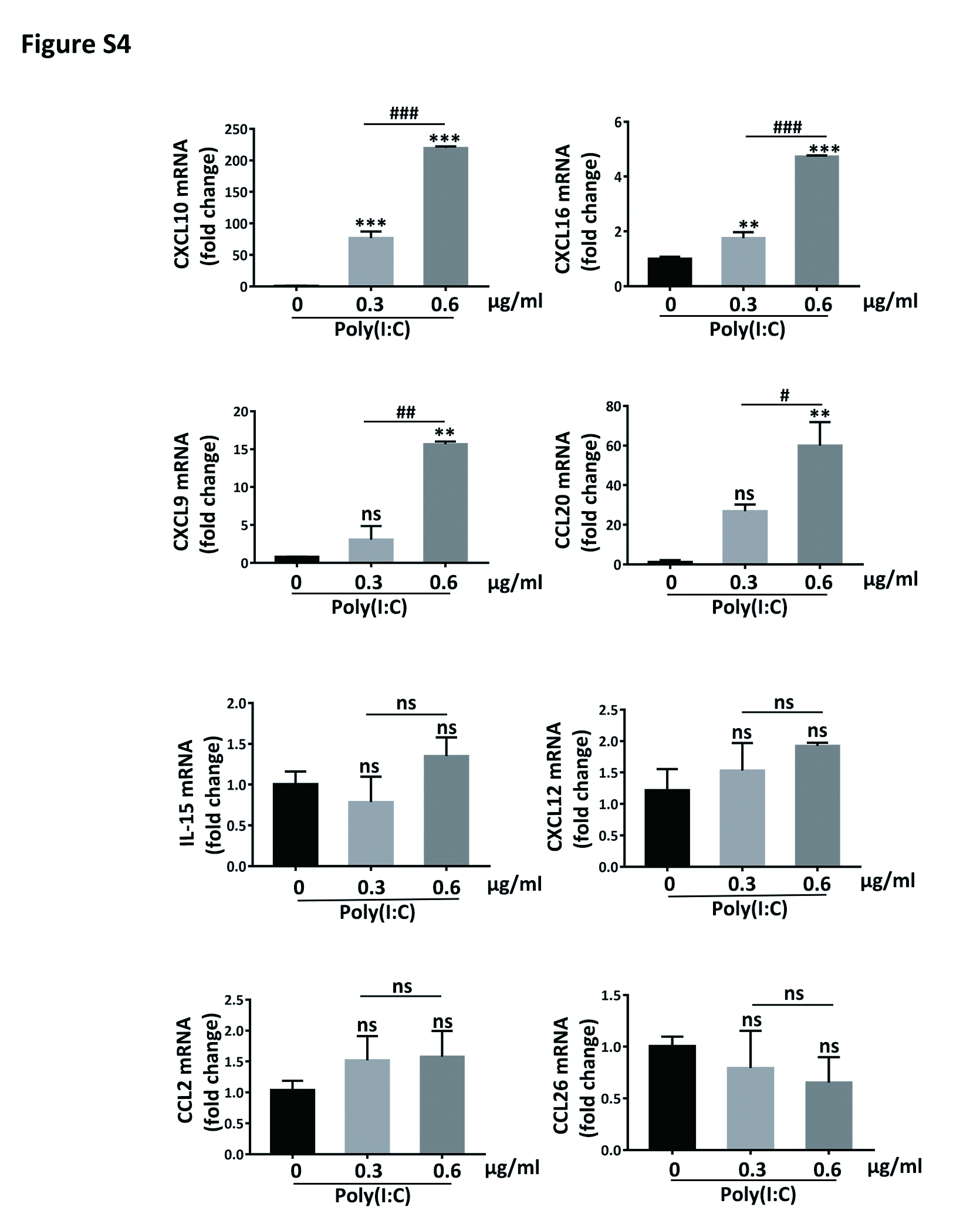

Supplement: Supplementary file 5 — Fig. S4 [file 41419_2020_2665_MOESM5_ESM.tif]

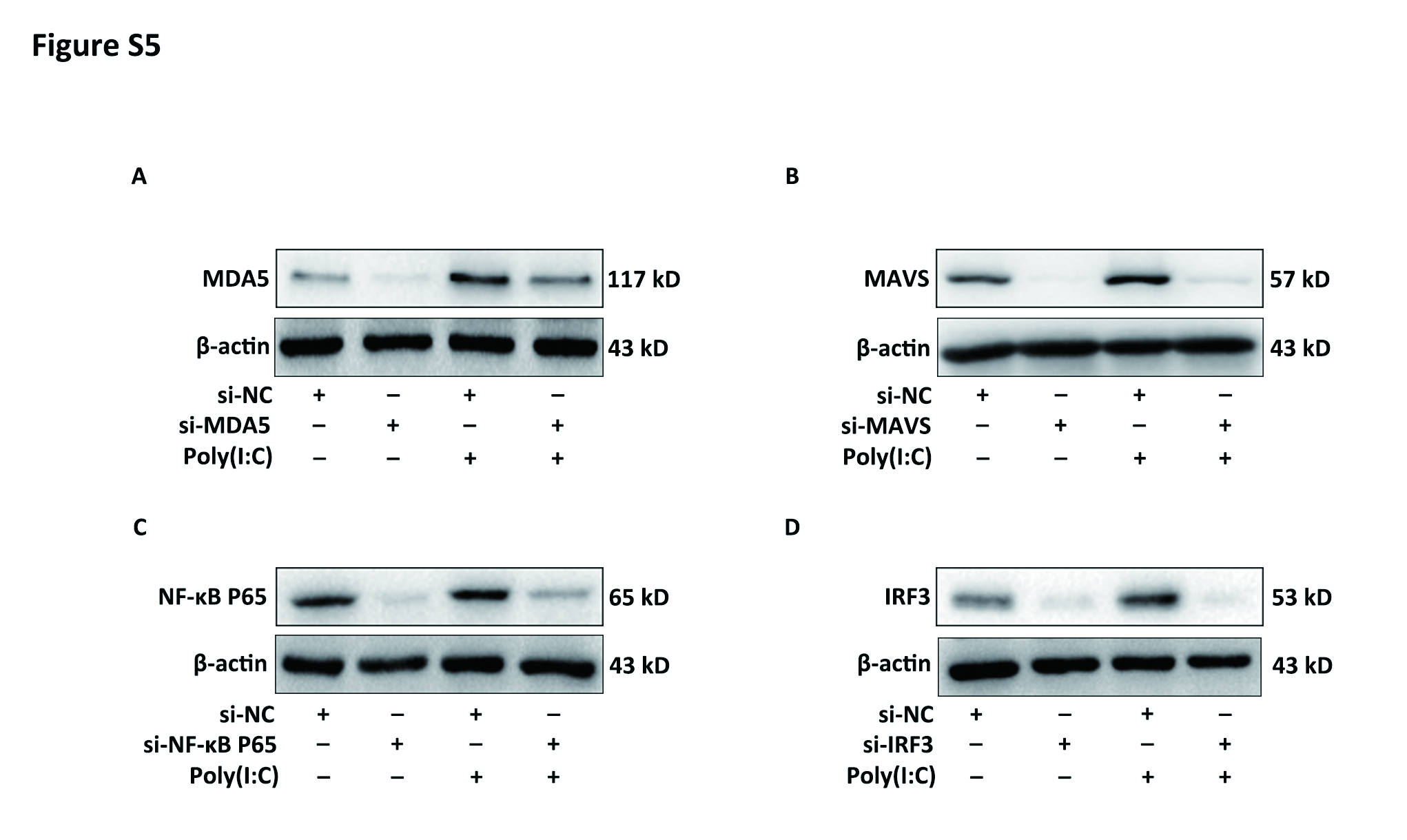

Supplement: Supplementary file 6 — Fig. S5 [file 41419_2020_2665_MOESM6_ESM.tif]

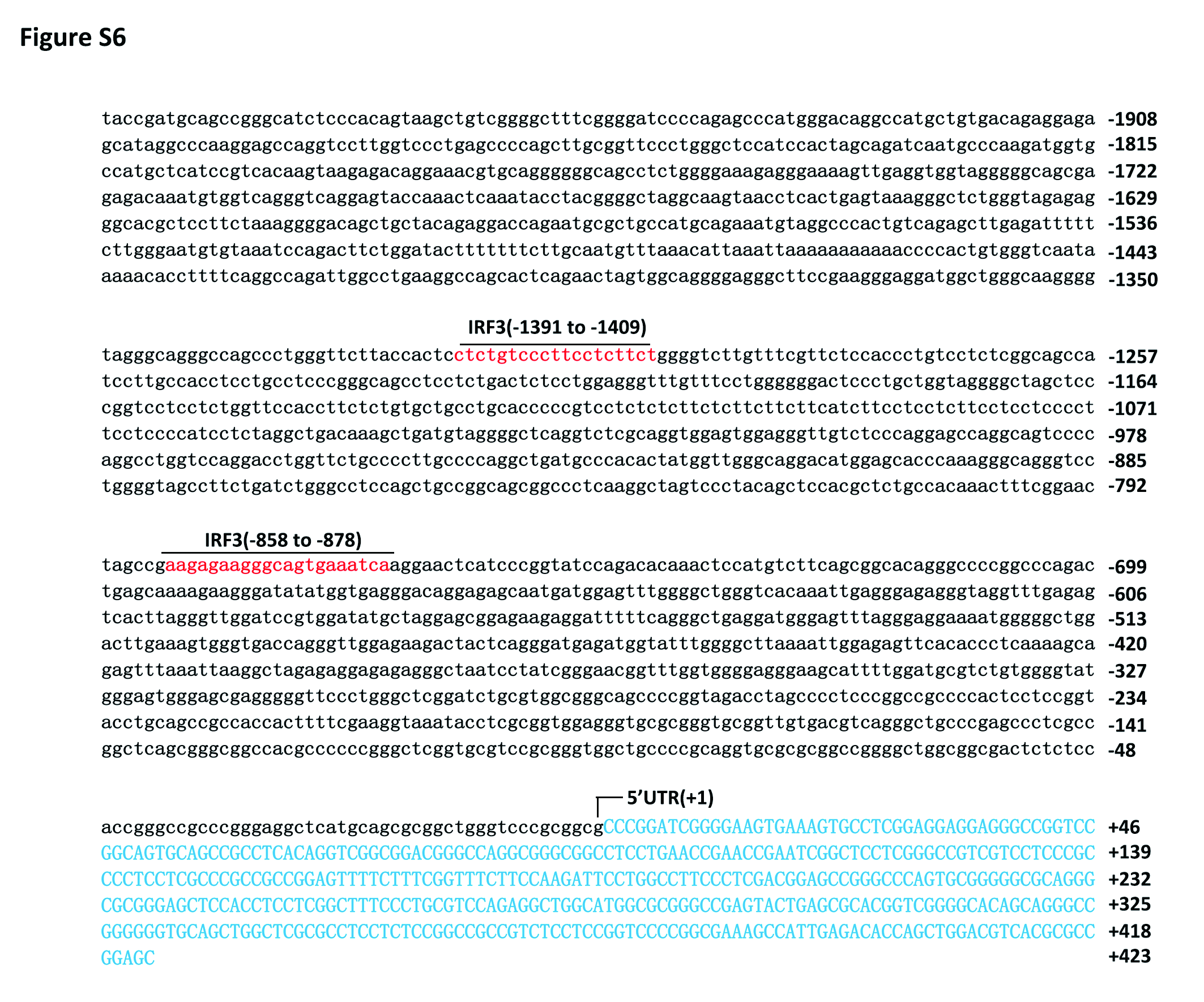

Supplement: Supplementary file 7 — Fig. S6 [file 41419_2020_2665_MOESM7_ESM.tif]
